# TRIM5 alpha Drives SIVsmm Evolution in Rhesus Macaques

**DOI:** 10.1371/journal.ppat.1003577

**Published:** 2013-08-22

**Authors:** Fan Wu, Andrea Kirmaier, Robert Goeken, Ilnour Ourmanov, Laura Hall, Jennifer S. Morgan, Kenta Matsuda, Alicia Buckler-White, Keiko Tomioka, Ronald Plishka, Sonya Whitted, Welkin Johnson, Vanessa M. Hirsch

**Affiliations:** 1 Laboratory of Molecular Microbiology, National Institute of Allergy and Infectious Diseases, National Institutes of Health, Bethesda, Maryland, United States of America; 2 Biology Department, Boston College, Chestnut Hill, Massachusetts, United States of America; SAIC-Frederick, United States of America

## Abstract

The antagonistic interaction with host restriction proteins is a major driver of evolutionary change for viruses. We previously reported that polymorphisms of the TRIM5α B30.2/SPRY domain impacted the level of SIVsmm viremia in rhesus macaques. Viremia in macaques homozygous for the non-restrictive TRIM5α allele TRIM5^Q^ was significantly higher than in macaques expressing two restrictive TRIM5alpha alleles TRIM5^TFP/TFP^ or TRIM5^Cyp/TFP^. Using this model, we observed that despite an early impact on viremia, SIVsmm overcame TRIM5α restriction at later stages of infection and that increasing viremia was associated with specific amino acid substitutions in capsid. Two amino acid substitutions (P37S and R98S) in the capsid region were associated with escape from TRIM5^TFP^ restriction and substitutions in the CypA binding-loop (GPLPA87-91) in capsid were associated with escape from TRIM5^Cyp^. Introduction of these mutations into the original SIVsmE543 clone not only resulted in escape from TRIM5α restriction in vitro but the P37S and R98S substitutions improved virus fitness in macaques with homozygous restrictive TRIM^TFP^ alleles in vivo. Similar substitutions were observed in other SIVsmm strains following transmission and passage in macaques, collectively providing direct evidence that TRIM5α exerts selective pressure on the cross-species transmission of SIV in primates.

## Author Summary

Human immunodeficiency virus (HIV) resulted from the transmission of simian immunodeficiency viruses (SIV) from nonhuman primates followed by adaptation and expansion as a pandemic in humans. This required the virus to overcome a variety of intrinsic host restriction factors in humans in order to replicate efficiently. Similarly, SIV encounters restriction factors upon cross-species transmission between nonhuman primates, specifically from a natural host species such as sooty mangabey monkeys to rhesus macaques. Previously we observed significant differences in the levels of virus replication of SIV among rhesus macaques due to subtle differences in one of these restriction factors, TRIM5 among individual macaques. Although a restrictive version of TRIM5 resulted in lower viremia, we also observed that the virus spontaneously mutated in the viral capsid gene and that these mutations were associated with escape from TRIM5 restriction. In the present study, we found that introduction of these escape mutations into the parental virus confers resistance to TRIM5 both in tissue culture and in macaques. These studies provide direct evidence that TRIM5 is a critical factor influencing the cross-species transmission of SIV in primates.

## Introduction

The epidemic of human immunodeficiency virus (HIV), including both HIV-1 and HIV-2, is a consequence of cross-species transmission of lentiviruses from non-human primates (NHP) to humans 1,2. HIV-1 is derived from cross-species infection of simian immunodeficiency virus in chimpanzees (SIVcpz) and HIV-2 from SIV in sooty mangabeys (SIVsmm) [3], [4], [5], [6]. The cross-species transmissions of SIV were also observed between primates of different species in the wild [7], [8], [9]. However, not all cross transmissions will result in epidemic infection in the new species. For HIV-1, several cross-transmission events, which occurred independently, generated the different distinct lineages, termed groups M, N, O and P, but only group M resulted in the worldwide pandemic of acquired immune deficiency syndrome (AIDS) in humans. For HIV-2, at least eight distinct lineages, termed groups A–H, were generated by independent cross-transmission, and only groups A and B have spread in the human population [2]. The divergence of several host proteins, including apolipoprotein B mRNA-editing, enzyme-catalytic, polypeptide-like 3G (APOBEC3G), Tetherin/BST-2, tripartite motif-containing protein 5α (TRIM5α) and SAM domain and HD domain-containing protein 1 (SAMHD1), constitute the specific restrictions preventing lentivirus cross-transmission among primates of different species [2], [10]. Only the virus strains which escape these restrictions are able to establish epidemic infection in a new host. The evolution and selection by interaction between viruses and host restriction factors resulted in the appearance of species-specific lentiviral lineages infecting different primates. Studies on how HIV/SIV interacts with restriction factors and overcomes the species specific barrier will not only help us to trace the origin of HIV/SIV, but also help us to understand the pathogenesis of HIV-1 infection. Such knowledge provides useful information for the development of anti-HIV drugs and vaccines. In our study, we used the SIVsmm-infected rhesus macaque model to study the relation between TRIM5α and SIV infection.

TRIM5α was first identified as a protein responsible for restriction of HIV-1 replication in macaque cell lines [11]. It is widely found and described as a retrovirus inhibitory protein in primates and several other mammals [11], [12], [13], [14], [15], [16], [17], [18], [19]. TRIM5α is a member of the tripartite motif or TRIM family of proteins which have RING finger, B-box, and coiled-coil structure domains. In addition to these three common domains shared by all TRIM family proteins, TRIM5α also has a B30.2/SPRY domain at its C terminus [20]. In some primate species, the TRIM5α B30.2/SPRY domains are replaced by cyclophilin-A (CypA) due to alternative mRNA splicing and these TRIM-Cyp variants also have restrictive activity against some retroviruses [14], [16], [21], [22], [23], [24], [25]. TRIM5α blocks lentivirus replication at a post-entry stage before reverse transcription [11], [26]. The detailed mechanism of TRIM5α restriction of lentivirus replication has not been well elucidated due to a lack of good methods for studying virus replication at this stage. However, binding to viral capsid protein is required for TRIM5α mediated restriction [26], [27], [28]. Several studies revealed that interaction between TRIM5α and lentiviral capsid protein resulted in dissociation of the viral core particle before reverse transcription [29], [30], [31], [32], [33], and triggering of the innate immune responses to restrict virus replication [34]. All the domains of TRIM5α are required for retroviral restriction activity and the B30.2/SPRY domain determines the specificity of capsid recognition and retroviral restriction [35], [36], [37], [38], [39]. Phylogenetic analysis of TRIM5α sequences from human and other species of NHPs indicated considerable interspecies variability and positive selection in the B30.2/SPRY domain, which revealed the important role of TRIM5α in fighting against virus infection during the evolutionary history of human and NHPs [18], [40], [41], [42].

In addition to interspecies variability, intra-species polymorphisms of TRIM5α were also reported in humans [43], [44], [45], [46], rhesus macaques and sooty mangabeys [47], [48]. In rhesus macaques, an insertion/deletion polymorphism at amino acid 339–341 of the B30.2/SPRY domain resulting in TFP/Q polymorphisms confers differential restriction against multiple lentiviruses when tested in a single-cycle infectivity assay [47], [48]. Five distinct alleles of TRIM5 have been described in macaques (Mamu-1 through Mamu-5). Mamu-1, 2 and 3 encode a TFP polymorphism in the SPRY domain and are restrictive for SIVsmm-infection, whereas, Mamu-4 and 5 encode a Q at this position and are permissive for SIVsmm infection [48]. A TRIM-CypA splice variant is also observed at a fairly low frequency in the rhesus populations and is also restrictive for SIVsmm-infection. The restrictive genotypes, TRIM5^TFP/TFP^, and TRIM5 ^TFP/Cyp^ and TRIM5^Cyp/Cyp^ represent 31, 10 and 2% respectively of a large cohort of rhesus macaques [unpublished data, WEJ, JSM] that have been genotyped for TRIM 5 (n = 1742). In our previous study, we found that these TFP/Q polymorphisms also affected SIVsmm replication in vivo. Viral loads in macaques with restrictive TRIM5^TFP/TFP^ and TRIM5 ^TFP/Cyp^ genotypes are approximately 100- to 1,000-fold lower than in macaques with permissive TRIM5α Q alleles [49]. Modest association of TRIM5α genotypes and infection was also reported in SIVmac251-infected rhesus cohorts, though the magnitude was smaller than what we observed in SIVsmE543-3 infected cohorts (∼1.3 log) [50]. SIVsmE543-3 has a history of only two passages in rhesus macaques after its initial isolation from sooty mangabeys [51], and thus it may not be completely adapted for replication in rhesus macaques. This contrasts with the repeated passage of SIVmac in rhesus macaques that has resulted in significant adaptation. Hence SIVsmE543-3 infected rhesus cohorts with different TRIM5α genotypes provide us a good model to study how SIV can overcome TRIM5α restriction.

## Results

### SIVsmm overcame TRIM5α restriction at late stages of infection of macaques

**Figure 1:**
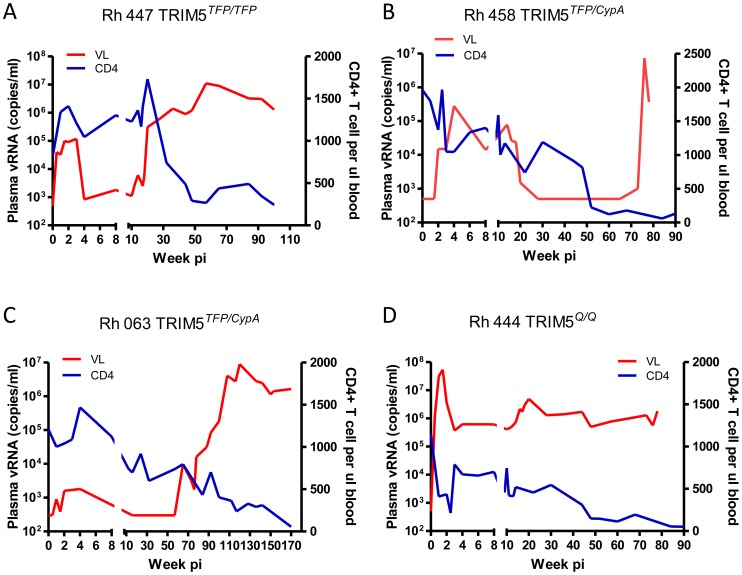
Replication of SIVsmE543-3 in rhesus macaques with different TRIM5 alleles. Viral loads (red lines) were quantified and shown as RNA copies in plasma samples from rhesus macaques Rh447 (A, TRIM5^TFP/TFP^), Rh458 (B, TRIM5^TFP/CypA^), Rh063 (C, TRIM5 ^TFP/CypA^) and Rh444 (D, TRIM5^Q/Q^). Peripheral CD4^+^ T cells (blue lines) were quantified by FACS and shown as the absolute numbers per microliter of blood.

To investigate whether SIV could overcome TRIM5α restriction, we monitored the plasma viremia and peripheral blood CD4+ T cell counts of four SIVsmE543-3-infected rhesus macaques that had different TRIM5α genotypes. All of these four macaques were intravenously inoculated with a high dose of SIVsmE543-3 as previously described [52]. Of these four macaques, one (Rh447) was homozygous for the restrictive TRIM5^TFP^ allele, two (Rh458 and Rh063) were heterozygous for the restrictive TRIM5^TFP^ and TRIM5^CypA^ alleles, and one (Rh444) was homozygous for the permissive TRIM5^Q^ allele. The macaques with the restrictive TRIM5^TFP/TFP^ (Rh447) and TRIM5^TFP/CypA^ (Rh458 and Rh063) genotypes had much lower plasma viral loads than the macaque with the permissive TRIM5^Q/Q^ genotype (Rh444) during the acute stage of infection (Fig. 1). The peak plasma viremia at the acute stage of infection varied from 10^3^ to 10^5^ copies per ml in these three macaques (Rh447, Rh458 and Rh063), at least two logs lower than in the permissive macaque Rh444. All three macaques with restrictive alleles maintained stable peripheral blood CD4+ T cell counts during acute infection (above 1000 CD4+ T cells per µl blood), while a significant loss of CD4+ T cells was observed in macaque Rh444. These results were consistent with a previous report, which revealed that TRIM5^TFP^ and TRIM5^CypA^ alleles restrict SIVsmE543-3 replication in rhesus macaques [49]. However, we also observed that at a later stage of infection, plasma viral loads in macaques Rh447, Rh458 and Rh063 increased to 10^6^ to 10^7^ copies per ml, which was comparable to that observed in the permissive macaque Rh444. Increasing viremia was accompanied by a significant loss of CD4+ T cells (less than 300 CD4+ T cells per µl blood). All the macaques progressed to AIDS with opportunistic infections within two to three years post infection despite the differences in their TRIM5α genotypes. These results suggested to us that SIV may have escaped TRIM5^TFP^ and TRIM5^CypA^ restriction at late stages of infection.

**Figure 2:**
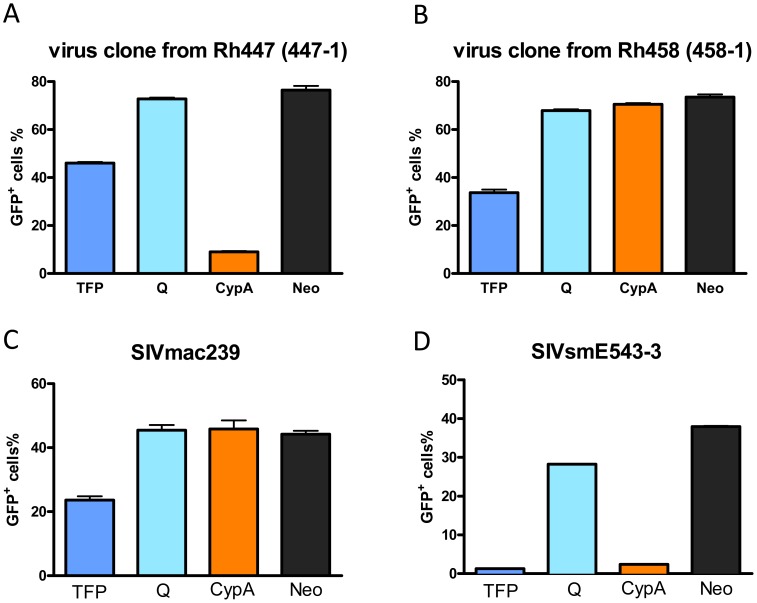
SIV clones from macaques with restrictive TRIM5 alleles overcame restriction at later stage of infection. Single-cycle infectivity of SIV clones from Rh447 (A, TRIM5^TFP/TFP^) and Rh458 (B, TRIM5^TFP/CypA^) was measured on a panel of cell lines stably expressing the rhesus TRIM5^TFP^ allele (dark blue bars), TRIM5^Q^ allele (light blue bars) and TRIM5^CypA^ (orange bars). Infectivity was measured as percent GFP positive cells. Black bars are negative vector controls. SIV clones SIVmac239 (C) and SIVsmE543-3 (D) were used as controls.

To confirm this hypothesis, we isolated SIV RNA from plasma samples of macaques Rh447 (at 78 w.p.i., weeks post infection) and Rh458 (at 110 w.p.i.), and obtained 8.5 kb RT-PCR products including the full gag-pol-env coding region. The PCR products were subcloned into the SIVsmE543-3 LTR backbone to construct full-length virus clones. Infectivity of these virus clones was measured on cell lines stably expressing different rhesus TRIM5α alleles by single-cycle infectivity assay as previously described [49]. As shown in Fig. 2, a representative virus clone, 447-1 from macaque Rh447 (TRIM5^TFP/TFP^) replicated in cell lines expressing TRIM5^TFP^ or TRIM5^Q^ alleles but was still restricted by the TRIM5^CypA^ allele. A virus clone, 458-1 from macaque Rh458 (TRIM5^TFP/CypA^) was able to replicate in cell lines expressing any of the alleles. Several other clones from Rh447 and Rh458 were tested and similar results were obtained (data not shown). In contrast, SIVsmE543-3 was restricted by the TRIM5^TFP^ and TRIM5^CypA^ alleles while SIVmac239 was resistant to all the alleles in the assay, which was consistent with the previous report [49]. These results confirmed that virus from late stage plasma had escaped TRIM5α restriction. Furthermore, the escape was specific to the restrictive alleles expressed by the macaques from which they were derived. Thus clones from Rh447 (TRIM5^TFP/TFP^) only escaped TRIM5^TFP^ restriction while clones from Rh458 (TRIM5^TFP/CypA^) escaped both TRIM5^TFP^ and TRIM5^CypA^ restriction, suggesting that the appearance of escape virus clones is due to the specific selection of TRIM5 restriction *in vivo*.

### Amino acid substitutions in capsid associated with SIVsmm escape from TRIM5α restriction

**Figure 3:**
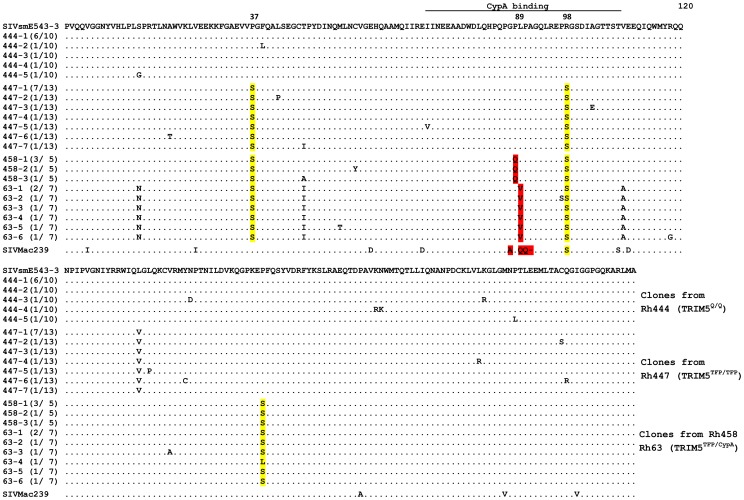
Identification of amino acid substitutions associated with escape from TRIM5 restriction. The capsid amino acids of SIV clones from Rh444 (TRIM5^Q/Q^), Rh447 (TRIM5^TFP/TFP^), Rh458 (TRIM5^TFP/CypA^) and Rh063 (TRIM5^TFP/CypA^) were aligned to parental SIVsmE543-3. Identical amino acids were shown as dot (.), deletions are shown as dash (-). Amino acid substitutions shared among SIV clones from different macaques were highlighted with yellow. Amino acid substitutions in the CypA binding loop are highlighted in red. The critical amino acid residues identified as responsible for escape from TRIM restriction are indicated by numbers above the sequence.

To further investigate how SIVsmm overcame TRIM5 restriction in these animals, we isolated SIV RNA from plasma samples sequentially collected from Rh444, Rh447, Rh458 and Rh063 at different time points during the course of infection (Fig. 1), cloned and sequenced the full gag coding regions. The capsid sequences were aligned and compared to the capsid sequences from the full length clones described above (447-1 and 458-1) and SIVsmE543-3 as shown in Fig. 3. In the permissive macaque Rh444 (TRIM5^Q/Q^), 4 of 10 clones had random amino acid substitutions and the other clones were identical to SIVsmE543-3 in capsid sequence. In contrast, common amino acid substitutions, “P37S”, “R98S” and “L135V” were found in capsid sequences of 13 clones from macaque Rh447 (TRIM5^TFP/TFP^). “P37S” and “R98S” were also found in all the clones from macaques Rh458 and Rh063 (TRIM5^TFP/CypA^). Clones from Rh458 and Rh063 had amino acid substitutions in the “GPLPA87-91” region, which is the site of CypA binding. Most of the clones from Rh458 and Rh063 also had a “P159S” substitution which is located at the C-terminal domain of the capsid protein. The capsid sequences were also compared to SIVmac239, which is not restricted by any of the rhesus TRIM5α alleles. SIVmac239 has mutations and a deletion, with “LPA” substituted by “QQ” in the CypA binding loop and a “R97S” (equivalent to R98S in SIVsmE543-3) substitution, consistent with important roles of these mutations in escaping from TRIM5α restriction, but does not have the P37S substitution.

**Figure 4:**
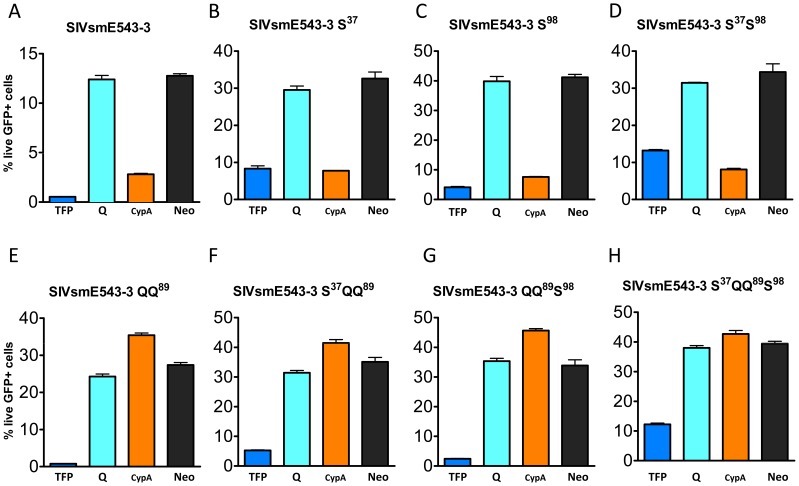
Introduction of amino acid substitutions into SIVsmE543-3 capsid conferred virus resistance to TRIM5 restriction. Single or combinations of amino acid substitutions “P37S”, “LPA89QQ” and “R98S” were introduced into SIVsmE543-3 capsid. Single-cycle infectivity of these mutants was measured on a panel of cell lines stably expressing a TRIM5^TFP^ allele (Mamu-2, dark blue bars), TRIM5^Q^ alleles (Mamu-4, light blue bars) and TRIM5^CypA^ (orange bars). Infectivity was measured as percent GFP positive cells. Black bars are negative vector controls. Infectivity on this panel are shown for SIVsmE543-3 (**A**), SIVsmE543-3 S^37^ (**B**), SIVsmE543-3 S^98^ (**C**), SIVsmE5433-3 S^37^ S^98^ (**D**), SIVsmE543-3 QQ^89^ (**E**), SIVsmE543-3 S^37^ QQ^89^ (**F**), SIVsmE543-3 QQ^89^ S^98^ (**G**) and SIVsmE543-3 S^37^ QQ^89^ S^98^ (**H**).

To confirm whether these substitutions conferred virus escape from TRIM5 restriction, we introduced the observed spontaneous mutations that appeared to be associated with escape from TRIM^TFP^ (P37S and R98S) into the SIVsmE543-3 capsid and constructed a series of mutants carrying single or a combination of these substitutions (Fig. 4 A–D). Their infectivity was measured on cell lines stably expressing a representative rhesus TRIM5^TFP^ allele (Mamu-2), a representative TRIM5^Q^ allele (Mamu-4) and the one TRIM5^CypA^ allele, as shown and listed in Fig. 4. While SIVsmE543-3 WT was restricted by both the TFP and CypA alleles, introduction of the P37S mutation resulted in some improvement in infectivity (Fig. 4B) while introduction of the R98S substitution had no effect on infectivity (Fig. 4C; p>0.05, Fig. S1). Introduction of both in combination (SIVsmE543-3 S^37^S^98^ (Fig. 4D) significantly improved infectivity relative to wild type (p<0.001, Fig. S1) to levels similar to SIVmac239. These results indicated that both the “P37S” and “R98S” substitutions are required for escape from TRIM5^TFP^ restriction in the context of the SIVsmE543-3 capsid. The requirement for the two substitutions was unexpected since SIVmac239 which is permissive for TRIM^TFP^ only has the equivalent of the R98S substitution. As predicted from our sequence analysis of escape variants in macaques, the P37S and R98S substitutions had no effect on TRIM^Cyp^ restriction.

We then focused on substitutions in the CypA binding loop, initially introducing the “LPA89QQ” substitution observed in SIVmac239 since SIVmac239was not restricted by TRIM5^CypA^ [49]. Introduction of “LPA89QQ” into SIVsmE543-3 greatly improved its infectivity on the cell line expressing the TRIM5^CypA^ allele (Fig. 4E, p<0.001, Figure S1) but had no effect on infectivity in the TRIM5^TFP^ cell line. The mutant SIVsmE543-3 QQ^98^ replicated on the TRIM5^CypA^ cell line as well as on the control cell line that lacked TRIM5α expression. We also examined the LPA89QQ substitution in combination with the two substitutions we had identified as critical for escape from TRIM^TFP^ (Fig. 4F, G and H). All the mutants carrying other substitutions combined with “LPA89QQ”, including E543-3 S^37^QQ^89^, E543-3QQ^89^S^98^, and E543-3 S^37^QQ^89^S^98^, also replicated well in the TRIM5^CypA^ cell line. These results indicated that the “LPA89QQ” substitution is sufficient to confer virus escape from TRIM5^CypA^ restriction. As expected both the P37S and R98S substitutions were required to confer escape to TRIM^TFP^ in the context of the CypA binding loop changes (Fig. 4H). Introduction of the spontaneous substitution in the CypA binding loop that we observed in our TRIM^TFP/CypA^ macaque, H458 (G87Q) also resulted in a significant improvement in infectivity in the TRIM^CypA^ cell line (data not shown) indicating that there are multiple avenues to achieve resistance to TRIM^CypA^. As expected, the mutant E543-3 S^37^QQ^89^S^98^, which carried the combination of substitutions “P37S”, “R98S” and “LPA89QQ”, was able to replicate on cell lines expressing either TRIM5^TFP^ or TRIM5^CypA^ alleles. All of these mutants replicated well on cell lines expressing the permissive TRIM5^Q^ alleles. These results are summarized in Table 1 and confirmed that the amino acid substitutions carried by late-stage clones from macaques with TRIM5^TFP^ or TRIM5^CypA^ alleles helped the virus overcome TRIM5α restriction.

**Table 1:** Summary of virus mutants and TRIM5 resistance.

Virus	Gag Capsid Mutation			TRIM5 resistance
	37	87–91	98	
E543	P	GPLPA	R	-
E543 S^37^	S	GPLPA	R	-
E543 S^98^	P	GPLPA	S	-
E543 S^37^S^98^	S	GPLPA	S	TFP
E543 QQ^89^	P	GPQQ	R	CypA
E543 S^37^QQ^89^	S	GPQQ	R	CypA
E543QQ^89^S^98^	P	GPQQ	S	CypA
E543 S^37^QQ^89^S^98^	S	GPQQ	S	TFP/CypA

### Escape from TRIM5α restriction improved virus fitness in rhesus macaques with restrictive TRIM5α alleles

**Figure 5:**
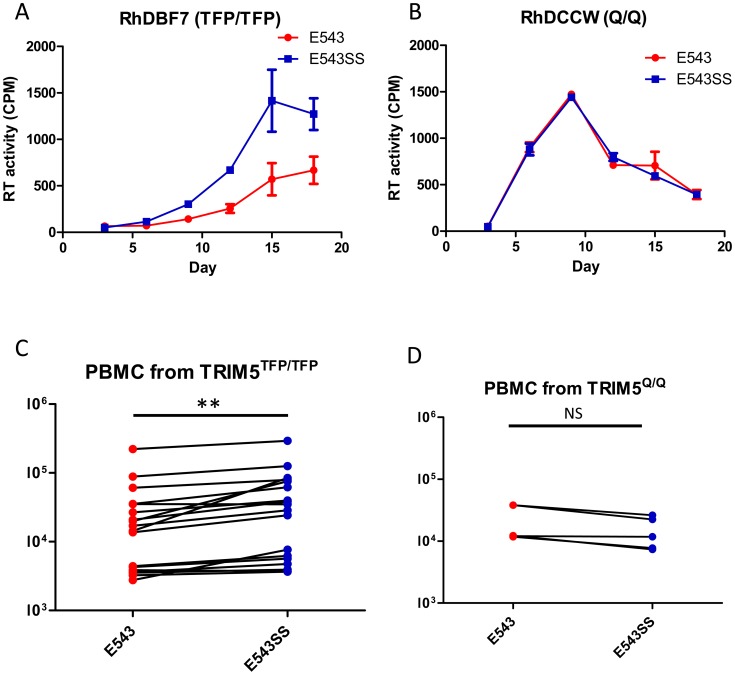
Comparison of PBMC infection with SIVsmE543-3 and SIVsmE543-3 S^37^S^98^. PBMCs were collected from 18 macaques with a TRIM5^TFP/TFP^ genotype and 5 macaques with a TRIM5^Q/Q^ genotype and activated with PHA for 3 days. Activated PBMCs were infected with SIVsmE543-3 and SIVsmE543-3 S^37^S^98^ at a M.O.I. of 0.001. Virus production was quantified and shown as RT values in supernatants collected at 3-day intervals. A and B show the replication kinetics of SIVsmE543-3 and SIVsmE543-3 S^37^S^98^ in PBMCs of representative macaques RhDBF7 (TRIM5^TFP/TFP^) and RhDCCW (TRIM5^Q/Q^). Area under the replication curve (AUC) of SIVsmE543-3 and the variant were calculated for each macaque separately. The AUC of SIVsmE543-3 and its variant in the TRIM5^TFP/TFP^ group (C) and the TRIM5^Q/Q^ group (D) are shown and compared by paired t test.

We further investigated whether the escape from TRIM5 restriction improved virus fitness in rhesus macaques that expressed restrictive TRIM5 alleles. Due to the low frequency of the TRIM5^CypA^ allele in rhesus macaque populations, we did not have sufficient macaques that were homozygous for the TRIM5^CypA^ allele for *in vivo* studies. Hence we only compared the replication of SIVsmE543-3 and SIVsmE543-3 S^37^S^98^, the mutant which carries both the “P37S” and “R98S” substitutions, in TRIM5^TFP^-homozygous macaques. Before inoculation into macaques, we investigated virus replication in cultured PBMC. After activation with phytohemagglutinin (PHA), PBMC from 18 macaques with a TRIM5^TFP/TFP^ genotype were infected with SIVsmE543-3 and SIVsmE543-3 S^37^S^98^ at a multiplicity of infection (M.O.I.) of 0.001. PBMC from five macaques with a TRIM5^Q/Q^ genotype served as positive controls. Virus production was measured by monitoring reverse transcriptase (RT) activity of supernatant collected at 3-day intervals and quantified with the phosphor imaging. Fig. 5 A and B show the replication kinetics of wild type and the variant SIV in PBMC of a representative TRIM5^TFP/TFP^ and TRIM5^Q/Q^ macaque, respectively. In PBMC from macaque RhDCWW (TRIM5^Q/Q^), SIVsmE543-3 replicated as well as mutant SIVsmE543-3 S^37^S^98^. Meanwhile in PBMC from macaque RhDBF7 (TRIM5^TFP/TFP^), the mutant replicated better than wild type SIVsmE543-3. To make a quantitative comparison, the area under the replication curve (AUC), which is indicative of total amount of virus production during the culture, was calculated and compared pairwise between these two viruses. As shown in Fig. 5 C and D, the mutant SIVsmE543-3 S^37^S^98^ replicated better than wild type in 18 TRIM5^TFP/TFP^ macaques and the difference was significant (Paired T test, P<0.01). In contrast, the replication of the two viruses in 5 TRIM5^Q/Q^ macaque PBMC was not significantly different (Paired T test, P>0.05).

**Figure 6:**
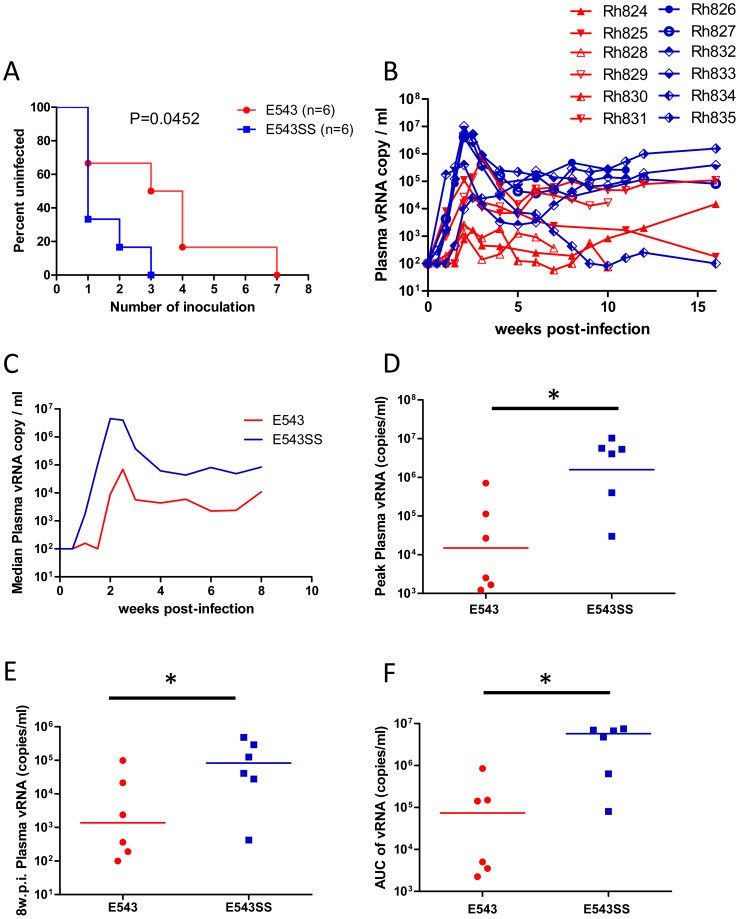
Acquisition and replication of SIVsmE543-3 and SIVsmE543-3 S^37^S^98^ in macaques with TRIM5^TFP/TFP^ genotype. Each macaque was inoculated intrarectally (I.R.) with 1000 TCID_50_ (5×10^5^ RNA copies of virus) and the infection was monitored by measuring plasma viral RNA load. Four weeks later any of the macaques that remained uninfected were inoculated intrarectally on a weekly schedule with same amount of virus until they became infected. The acquisition of infection in each group was shown as uninfected percentage after each inoculation and compared by log-rank test. Median inoculation time was 3.5 for SIVsmE543-3 challenge group and 1 for SIVsmE543-3 S^37^S^98^ challenge group (A). Plasma viral RNA copies in each macaque (B) and median plasma viral RNA copies in each group (C) are shown. Peak plasma viral loads (D, P = 0.0152), plasma viral loads at 8 w.p.i. (E, P = 0.0411) and viral load AUC before 8 w.p.i. (F, P = 0.0260) were compared by non-parametric Mann-Whitney-test.

Then we compared the replication of these wild type SIVsmE543-3 and the putative TRIM^TFP^-resistant variant (SIVsmE543-3S^37^S^98^) *in vivo*. Twelve rhesus macaques with TRIM5^TFP/TFP^ genotypes were divided into two groups; relative *in vitro* infectivity of the variant in PBMC of the animals was not considered in selection. To minimize the effect of MHC restriction on virus load, the distribution of MHC I genotypes known to have an effect on SIVmac239 viremia was balanced between the two groups as shown in Table 2. Before inoculation, viruses were expanded on PBMC from the permissive TRIM5^Q/Q^ macaque, RhDCCW. Gag sequences of the two virus stocks were evaluated after expansion on PBMC and no additional mutations were found in either of the two virus stocks (data not shown). Each macaque was inoculated intrarectally (I.R.) with 1000 TCID_50_ (5×10^5^ RNA copies of virus) and the infection was evaluated by monitoring plasma viral RNA load. Four weeks later, any of the macaques that remained uninfected were inoculated intrarectally on a weekly schedule with the same amount of virus until they became infected. There was no correlation between expression of a known restrictive MHC-I genotype and either acquisition or viral load in acute phase of infection. We compared the acquisition of infection of the group receiving SIVsmE543-3 and SIVsmE543-3S^37^S^98^ by Kaplan-Meier curves as shown in Fig. 6A. In the group inoculated with the variant, SIVsmE543-3 S^37^S^98^, four of six macaques were infected after the first inoculation, while in the group inoculated with the wild type SIVsmE543-3 only two macaques were infected after the first inoculation. Macaques inoculated with SIVsmE543-3 S^37^S^98^ required significantly less exposure to get infected than macaques inoculated with SIVsmE543-3 (log-rank test, P = 0.0452). It required 3.5 inoculations to infect half of the macaques with SIVsmE543-3, while it only required one inoculation by SIVsmE543-3 S^37^S^98^. We also compared the plasma viral loads of these two groups after infection. The plasma viral loads for each macaque and the median plasma viral load for each group are shown in Fig. 6 B and C. Macaques infected with SIVsmE543-3 S^37^S^98^ had significantly higher plasma viremia compared with macaques infected with SIVsmE543-3, with mean differences of 105-fold at peak, and 25-fold at 8 w.p.i. As a measure of cumulative virus replication, we also compared the area under the curve (AUC) during the acute phase of infection (1–8 weeks) and observed a 70-fold higher level in macaques inoculated with the variant virus (Fig. 6 D,E and F). The *in vivo* infection results, combined with *in vitro* PBMC infection results, indicated that the “P37S” and “R98S” substitutions improved virus fitness in macaques with TRIM5^TFP/TFP^ genotypes, which also suggested that the appearance of variants carrying these mutations was due to TRIM5α selection.

**Table 2:** MHC genotype of macaques.

SIV virus	Macaque ID	MHC I
**SIVsmE543-3**	Rh824	A08, B01
	Rh825	A02, B01
	Rh828	**A01**, A08, **B17**
	Rh829	Negative
	Rh830	A08
	Rh831	**B17**
**SIVsmE543-3 S^37^S^98^**	Rh826	B01
	Rh827	A02, A08
	Rh832	**A01**, B01
	Rh833	A08, B01, **B08**
	Rh834	**B17**
	Rh835	Negative

Nine rhesus MHC class I alleles, including Mamu-A*001, A*002, A*008, A*011, B*001, B* 003, B*004, B*008, B*017, were tested and listed for each macaque. Negative: none of the 9 alleles observed. MHC I genotypes known to have an effect on SIVmac239 viremia are indicated in bold.

### TRIM5α exerted selective pressure on SIVsmm transmission into macaques

**Figure 7:**
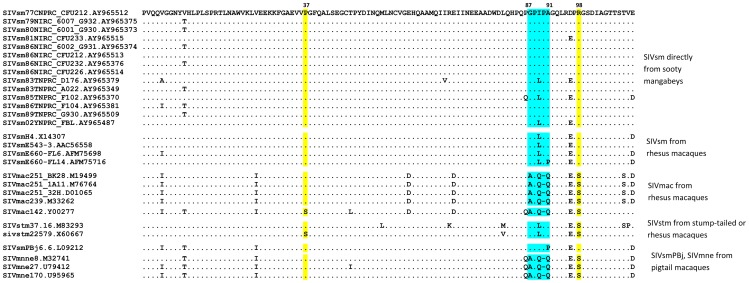
Variance of SIV capsid sequences is associated with their passage history in macaques. The capsid N-terminal domain of SIV clones, with or without passage in rhesus, stump-tailed and pigtail macaques, were aligned to a primary SIVsm clone from sooty mangabey (top). Identical amino acids were shown as a dot (.), deletions are shown as a dash (-). The sites under TRIM5 selection are highlighted in yellow and the Cyclophilin-A binding site is highlighted in light blue and the critical amino acid residues identified as responsible for escape from TRIM restriction are indicated by numbers above the sequence.

We were interested in determining whether the changes we observed in SIVsmE543 could be generalized to other SIVsmm/mac strains so we investigated the capsid sequences of SIV isolates commonly used in NHP models for evidence of TRIM5 selection. Four lineages of SIV strains, including SIVsmm, SIVmac, SIVstm and SIVmne, were isolated from infected macaques during several independent SIV transmission events in the 1970s to 1980s [4], [51], [53], [54], [55], [56], [57], [58], [59], [60], [61], [62], [63], [64]. The macaque passage history of commonly used SIV isolates is briefly summarized in Table 3 and the sequences of their capsid N-terminal domains are aligned and showed in Fig. 7. Phylogenetic analysis indicated that all of these SIV isolates originate from the cross-transmission of SIVsmm from sooty mangabeys, followed by unknown or intended experimental passages in different species including rhesus, pigtail and stump-tailed macaques [65], [66], [67]. Capsid sequences of primary clones of SIV directly isolated from sooty mangabey monkeys [66] were highly conserved. All SIVsmm clones from sooty mangabeys encoded a “P37” and “R98” in their capsid, similar to SIVsmE543-3. Some SIVsmm clones encoded an “I91L” variation in the CypA binding loop which does not affect sensitivity to TRIMCypA restriction [49]. The lack of variability in the sequence alignment of primary SIVsmm capsid proteins suggested that there was no TRIM5α restriction and selection during the passage of SIVsmm among sooty mangabeys. The limited passage of SIVsmm from the Tulane Primate Center in rhesus macaques resulted in the isolation of several SIVsmm clones including SIVsmH4, SIVsmE543-3, and SIVsmE660 clones FL6 and FL14 [4], [54], [68]. All of these clones, except for SIVsmE660-FL14, carried the“P37”, “R98” and “LPA89” observed in the capsids of primary SIVsmm clones. SIVsmE660-FL14 had a “A91P” substitution, which allowed escape from TRIM5^CypA^ restriction (data not shown), suggesting that the passage history of SIVsmE660 may have involved exposure to TRIM5^CypA^ selection. The long-term passage of SIVsmm in rhesus macaques resulted in the isolation of SIVmac [55], [69]. SIVmac251 clones and SIVmac239 had “LPA89QQ” and “R97S” (equivalent to R98S in SIVsmm) substitutions in their capsids. SIVmac142, which is independent of the SIVmac251/239 passage chain, had the “P37S” substitution in addition to the “LPA89QQ” and “R97S”substitutions. SIVstm underwent an unknown history of passage in stump-tailed macaques (*Macaca arctoides*) after the original SIVsmm cross-species transmission. Only two SIVstm clones are available. SIVstm37.16, which was directly cloned from an infected stump-tailed macaque [56], only has the “R98S” substitution in capsid. The other, SIVstm22579, isolated after two passages in rhesus macaques [57] has both the “P37S” and “R98S” in capsid identified as critical for escape from TRIM^TFP^ restriction but no changes in the GPLPA motif. Finally, SIVsmPBj which has a history of passage in pigtail macaques has a substitution in the CypA binding loop of capsid, “A91P” conferring virus escape from TRIM5^CypA^ restriction, which is consistent with pigtail macaques exclusively carrying the TIRM5^CypA^ allele [22]. The variance of these mutations in different SIV clones was associated with their different passage history in macaques, suggestive of TRIM5 selection during their cross-transmission and adaptation. However a common pathway of escape mutations similar to what we observed spontaneously in SIVsmE543 was observed in all of these adapted viruses.

**Table 3:** Passage history of SIV clones.

	SIV clones		*In vivo* macaque passage				Reference
	Clone Name	Accession Number	Sooty Mangabey	Stump-tail	Rhesus	Pig tail	
**SIVsm**	**SIVsmH4**	X14307	SM E038	NO	RhF236	NO	[4]
	**SIVsmE543**	U72748	SM E038	NO	RhF236 RhE543^2^	NO	[51]
	**SIVsmE660-FL6**	JQ864085	SM E038	NO	RhF236 RhE543 RhE660	NO	[54]
	**SIVsmE660-FL14**	JQ864087	SM E038	NO	RhF236 RhE543 RhE660	NO	[54]
**SIVmac** 1	**SIVmac251 1A11**	M76764	unknown	NO	Rh78 Rh251	NO	[55], [58]
	**SIVmac251 BK28**	M19499	unknown	NO	Rh78 Rh251	NO	[55]
	**SIVmac251 32H**	D01065	unknown	NO	Rh78 Rh251 Rh32H	NO	[53], [55]
	**SIVmac239**	M33262	unknown	NO	Rh78 Rh251 Rh61 Rh239	NO	[55], [59]
	**SIVmac142**	Y00277	unknown	NO	Rh142	NO	[55], [60]
**SIVstm**	**SIVstm37.16**	M83293	unknown	N.A.	NO	NO	[56]
	**SIVstm22579**	X60667	unknown	N.A.	Rh21685 Rh22579	NO	[57]
**SIVPBj**	**SIVPBj6.6**	L09212	unknown	NO	NO	PT PBj	[61]
**SIVmne**	**SIVmne08**	M32741	unknown	NO	NO	PT T76321	[62]
	**SIVmne27**	U79412	unknown	NO	NO	PT T76321PT T78027	[62], [63]
	**SIVmne170**	U95965	unknown	NO	NO	PT T76321PT T78027	[62], [64]

1 : For SIVmac, unrecorded passages in rhesus macaques may be involved before passage in the listed rhesus macaques.

2 . RhE543 is the only macaque with known TRIM5 genotype (TRIM5^Q/Q^).

## Discussion

The antagonistic interaction with host restriction proteins is considered a major driver of evolutionary change for viruses. We previously reported that the polymorphism of TRIM5α affected SIVsmm replication in rhesus macaques, which suggested that TRIM5α restriction also plays an important role in HIV/SIV evolution [49]. Here we showed that selection of TRIM5 resistant SIVsmm capsid mutants at late stages of infection in rhesus macaques expressing restrictive TRIM5 alleles. The resistance to TRIM5 restriction was associated with three common amino acid substitutions in the N-terminal domain of the capsid protein. By site-direct mutagenesis, we confirmed that these substitutions conferred TRIM5 restriction and improved the virus fitness in rhesus macaques with restrictive TRIM5 alleles. These results provide both *in vitro* and *in vivo* evidence to support the hypothesis that TRIM5 exerts selective pressure on SIV evolution.

Our results also demonstrate the value of using SIV infected rhesus macaques to study the influence of restriction proteins during HIV/SIV cross-transmission. Several reports have revealed that interactions between the host restriction proteins APOBEC3G and Tetherin/BST-2 with HIV/SIV resulted in the acquisition and/or evolution of the Vif, Vpu and Nef proteins [70], [71], [72], [73]. Since it is impossible to trace back the cross transmission events which occurred hundreds or thousands of years ago, most of these studies were based on phylogenetic analysis of host restriction genes and SIV sequences in different primate species followed by in vitro infection inhibition assays. The question remaining however is in what way the selection and adaptation occur. In this paper, macaques expressing restrictive TRIM5 alleles were infected by two different routes. Three macaques (Rh447, Rh458 and Rh063) were intravenously inoculated with a high dose of wild type SIVsmE543-3. For these macaques, the restrictive alleles did not prevent infection but the virus maintained a low level of replication until the appearance of mutations associated with TRIM5 resistance. The other twelve macaques were inoculated by a more biologically relevant mucosal method using repetitive lower dose intrarectal inoculation with the wild type SIVsmE543-3 or the TRIM5-resistant SIVsmE543-3S^37^S^98^ mutant. This allowed us to observe that the TRIM5-resistant mutant had enhanced acquisition of infection in addition to improved viral replication when compared to wild type virus.

To determine whether this was a phenomenon that could be generalized to all SIVsmm strains, we attempted to trace the influence of TRIM5 selection on the cross-species transmission of SIVsmm into captive macaques in the 1970s. The primary SIVsmm clones derived directly from sooty mangabeys showed a high degree of conservation in the capsid protein despite being isolated from separate mangabeys in different U.S. national primate centers or from wild caught animals in West Africa [66]. None of the SIVsmm clones had any of the amino acid substitutions that have been associated with escape from TRIM5α restriction. Since all evidence suggests that SIVsmm has circulated among sooty mangabeys for quite a long time, it may be completely adapted to this host and not restricted by sooty TRIM5α. Polymorphism of TRIM5α has also reported in sooty mangabeys and four different alleles have been identified [47]. However, none of these sooty TRIM5α alleles restricted SIVsmE543-3 replication in single-cycle infectivity assays (data not shown) consistent with the long-term co-evolution of this virus in this species. It also suggests that TRIM5 restriction and selection occurred mainly at the time when SIV was being introduced into a new primate species. When compared with primary SIVsmm clones from sooty mangabeys, many SIV clones that evolved in macaques following experimental passage, including SIVmac, SIVsmm, SIVstm and SIVmne, had amino acid substitutions/deletions at the sites under TRIM5 selection in capsid. The mutations varied depending on their passage history in macaques. Although there is no record to trace exactly when and how SIVsmm was introduced into captive macaques, the sequence alignment of SIV capsid of these clones revealed that the TRIM5 genotypes of the macaques during virus passage probably also exerted selective pressure on SIV evolution in macaques. SIVsmE543 had only been passaged through two rhesus macaques and at least one of these rhesus was homozygous permissive for TRIM5 [49], which is consistent with the lack of escape substitutions in the capsid of this virus. Other viruses exhibited a range of escape mutations, with viruses passaged the most extensively in rhesus macaques, such as SIVmac239, exhibiting escape from both restrictive alleles and others such as those passaged in pigtail macaques (and thus only subjected to TRIM^CypA^ selection), only having changes in the Cyclophylin A binding loop of capsid. Such a process of restriction and selection may also affect SIV infection in primates of other species, as is indicated by the observed variation of SIV capsid sequences from primates of different species [74]. However the amino acid sites associated with resistance to TRIM5 may be not the same as what we observed in SIVsmm-infected macaques and will require further study for identification.

Regarding the significance of TRIM5 polymorphisms in modulating HIV-infection of humans, TRIM5α polymorphism has also been reported in human populations. Several nonsynonymous SNPs were found in screening of more than 1000 samples from human populations. Two nonsynonymous SNPs, resulting in amino acid polymorphisms H43Y in the RING domain and R136Q in the coiled-coiled domain, were reported to affect the *in vitro* TRIM5α restriction of HIV-1 and were differently distributed in HIV-1 positive and negative cohorts [43], [44], [45], [46], [75]. However, whether these SNPs actually affect virus acquisition result is controversial since some groups suggested that the effect of TRIM5α polymorphisms on HIV-1 disease progression was minimal [46]. Since HIV-1 had already circulated in a small population in Africa for several decades before causing a worldwide pandemic in the 1980s [76], the virus may have already adapted and escaped from human TRIM5α restriction during early passage in humans. Furthermore, the TRIM5α sequences within human beings are much less divergent than within rhesus macaques and only few polymorphisms in human TRIM5α were located in the SPRY domain, which may be due to the lack of positive selection by other retroviruses during the evolution of humans [43]. The low variability of human TRIM5α may also explain the lack of restriction of human TRIM5α on HIV-1 replication.

Our studies also provide a model to study the mechanism of TRIM5α restriction. The conserved capsid protein is a potential target for anti-HIV drug development because of its important role in virus replication. Studies on interaction between TRIM5α and the HIV/SIV capsid will provide useful information for anti-HIV drug development. However, the mechanism of TRIM5α restriction on HIV/SIV replication remains unclear. A recent report by Pertel *et al.* indicated that interaction of TRIM5α with the retrovirus capsid lattice promotes innate immune signaling [34]. Another report by Battivelli *et al.* showed that the CTL escape mutants isolated from HIV-infected patients are more sensitive to human TRIM5α restriction than laboratory-adapted HIV-1 strains [77]. These reports revealed that TRIM5α restriction of retroviruses is not only mediated through capsid lattice dissociation and degradation brought on by interaction between TRIM5α and the capsid protein, but are also linked to innate and adaptive immune responses. Macaques infected with SIVsmE543-3 and its TRIM5-resistant variants will be a valuable animal model to study the mechanism of TRIM5 restriction *in vivo*.

Our study also has quite practical applications to improve the models of SIV-infected macaques for HIV-1 research. The present study focused on escape mutations for one of the most common restrictive TRIM5 alleles to determine whether the spontaneous substitutions were associated with biological escape from TRIM5 restriction in vivo. Obviously a virus that has escaped both restrictive alleles is still required as a challenge virus for vaccine trials. SIVsmE543-3, although being pathogenic, has not been widely used as a challenge strain and thus may not be the ideal target for these studies. SIVsmE660, which was isolated from a SIVsmE543 infected macaque, is widely used as a challenge stock in the rhesus macaque model for vaccine evaluation. Several groups reported that the replication of SIVsmE660 was also affected by TRIM5 alleles: these studies suggest that TRIM restriction of this virus can affect acquisition of infection in repetitive “low” dose intra-rectal challenge models [78], [79]. This effect can confound vaccine evaluation unless study groups are balanced for TRIM5 alleles or animals with restrictive alleles are excluded from the study. In our previous report, we described several infectious molecular clones from a SIVsmE660 virus stock and found that all of the clones were restricted by TRIM5^TFP^, and one SIVsmE660 clone had TRIM5^CypA^ escape mutations due to substitutions in the CypA binding loop of capsid [54]. The results in this paper suggest that the introduction of the “P37S” “R98S” substitutions into SIVsmE660 clones may confer escape from TRIM5^TFP^ restriction, and provide virus clones not restricted by TRIM5 for use as challenge viruses in vaccine trials. The replication and pathogenesis of SIVsmE660 clones with these mutations is under evaluation in rhesus macaques.

## Materials and Methods

### Ethics

This study was carried out in strict accordance with the recommendations described in the Guide for the Care and Use of Laboratory Animals of the National Institute of Health, the Office of Animal Welfare and the United States Department of Agriculture. All animal work was approved by the NIAID Division of Intramural Research Animal Care and Use Committees (IACUC), in Bethesda, MD (protocol # LMM-6. The animal facility is accredited by the American Association for Accreditation of Laboratory Animal Care. All procedures were carried out under Ketamine anesthesia by trained personnel under the supervision of veterinary staff and all efforts were made to ameliorate the welfare and to minimize animal suffering in accordance with the “Weatherall report for the use of non-human primates” recommendations. Animals were housed in adjoining individual primate cages allowing social interactions, under controlled conditions of humidity, temperature and light (12-hour light/12-hour dark cycles). Food and water were available *ad libitum*. Animals were monitored twice daily (pre- and post-challenge) and fed commercial monkey chow, treats and fruit twice daily by trained personnel. Early endpoint criteria, as specified by the IACUC approved score parameters, were used to determine when animals should be humanely euthanized.

### Animals

Colony-bred rhesus macaques of Indian origin (*Macaca mulatta*) were housed in a BSL2 facility using BSL3 practices. The TRIM5 genotype of rhesus macaques were determined as previously described [49], and MHC I genotypes were determined by the Rhesus Macaque MHC Typing Core facility at the University of Wisconsin. Four rhesus macaques (Rh444, Rh447, Rh458 and Rh063) were intravenously infected with the SIVsmE543-3 molecular clone as previously described [52]. Blood and plasma were collected sequentially. The viral RNA levels in plasma were determined by quantitative reverse transcriptase PCR (RT-PCR) and blood CD4+ T cell subsets were quantified by flow cytometric analysis as previously described [54].

### RT-PCR

Virus RNA was isolated from plasma of macaque Rh447 and Rh458 with the QiaAmp Viral RNA kit (QIAGEN, Germany). Reverse transcription of viral RNA to single-stranded cDNA was performed using the SuperScript III first-strand synthesis system (Invitrogen, Carlsbad, CA) with primer R-R (5′-TGC TTA CTT CTA AAT GGC AGC TTT-3) according to the manufacturer's instructions. Full-length gag-pol-env cassettes (including the entire gag, pol, vif, vpr, tat, rev, env genes and parts of the nef gene) were amplified by nested PCR using Platinum Taq Hi Fidelity (Invitrogen) polymerase. First-round PCR was performed by using 1 µl of bulk cDNA with primers Nar-F (5′-GGTTGGCGC CCG AAC AGG GAC TT-3′) and R-R under the following cycling conditions: 94°C for 2 min, followed by 35 cycles of 94°C for 30 s, 60°C for 30 s, and 68°C for 9 min, with a final extension of 68°C for 10 min. Second-round PCR was performed by using 1 µl of the first-round PCR product with primers Nar-F and Bgl-R (5′-GGC CAA GAT CTG CTG CCA CCT CTG TC-3′) under the same conditions used for the first-round PCR. PCR products were subcloned into SIVsmE543-3 by cutting with restriction enzymes NarI and BglII to construct chimeric virus clones.

To sequence the gag gene, virus RNA was isolated from plasma of macaque Rh444, Rh447, Rh458 and Rh063. Reverse transcription was performed as described above. 1.8 kb PCR products covering the full gag region were amplified from 1 µl of bulk cDNA with primers Nar-F (5′-GGTTGGCGC CCG AAC AGG GAC TT-3′) and Gag-R (5′-GCT GAT GAT TCA ATT GTA ACA GG-3′) under following cycling conditions: 94°C for 2 min followed by 35 cycles of 94°C for 30 s, 60°C for 30 s, and 68°C for 2 min, with a final extension of 68°C for 5 min. PCR products were cloned into PCR4-Topo vector with TOPO-TA cloning kit (Invitrogen) and sequenced. All sequences were deposited in GenBank under the following accession numbers (KC904980-KC905000). Gag sequences were aligned using ClustalX2 and compared with the parental SIVsmE543-3 clone (GenBank accession U72748) and SIVmac239 (GenBank accession M33262).

### Site-directed mutagenesis

Amino acid substitutions “P37S”, “LPA89QQ” and “R98S” were introduced into SIVsmE543-3 capsid by using QuikChange II Site-Directed Mutagenesis Kits (Agilent) according to the manufacturer's instructions. The following primer sets were used to introduce mutations: P37S: forward: 5′- GGC AGA GGT AGT GTC AGG ATT TCA AGC G-3′; reverse: 5′- CGC TTG AAA TCC TGA CAC TAC CTC TGC C-3′; LPA89QQ: forward: 5′-CAG CCA GGT CCA CAA CAA GGG CAA C-3′; reverse: 5′-GTT GCC CTT GTT GTG GAC CTG GCT G-3′; R98S: forward: 5′-GCA ACT TAG AGA GCC ATC AGG ATC AGA CAT TGC AG-3′; reverse: 5′-CTG CAA TGT CTG ATC CTG ATG GCT CTC TAA GTT GC-3′;

### Single-cycle infectivity assay

Retroviral vector V1EGFP-SIV and Crandell-Rees Feline Kidney (CRFK) cell lines stably expressing six common rhesus TRIM5 alleles, including three TRIM5^TFP^ alleles, two TRIM5^Q^ alleles, and the TRIM5^CypA^ allele were described in a previous report [49]. The entire gag-pol-vif segment of SIVsmE543-3 mutants or virus clones from macaque Rh447 and Rh458 were subcloned into V1EGFP-SIV by cutting with restriction enzymes NarI and BstBI. Single-cycle SIV viruses were produced in 293T cells by co-transfection of V1EGFP-SIV and pVSV-G, titered and infectivity measured on CRFK cell lines stably expressing TRIM5 alleles as previous described [49].

### Preparation of virus stocks

293T cells were maintained in Gibco GlutaMAx DMEM media plus 10% FCS, 100 U/ml penicillin, and 100 µg/ml streptomycin and transfected with 10 µg SIVsmE543-3 mutants plasmid using FuGENE 6 transfection reagent (Roche Diagnostics, Indianapolis, IN). Virus stocks were collected from the supernatant of transfected cells after 48 hours and filtered through a 0.22 um filter. The tissue culture infectivity dose (TCID_50_) of virus stocks was tested on TZM-bl cells and calculated by the Reed & Muench method [80].

Viruses were expanded on rhesus PBMCs before inoculation into rhesus macaques. PBMCs were collected from a permissive TRIM5^Q/Q^ macaque, RhDCCW and cultured in complete RPMI 1640 media containing 10% interleukin-2 (IL-2) After stimulation with 2 µg/ml phytohemagglutinin (PHA) for 72 hours, activated PBMC were infected with SIVsmE543-3 and SIVsmE543-3 S^37^S^98^, respectively, at an M.O.I. of 0.001. Infected PBMCs were washed and cultured in complete RPMI 1640 media containing 10% IL-2. Virus stocks were collected at day six and filtered, followed by titration on TZM-bl cells and quantitation by real-time RT-PCR. The gag regions were amplified from virus PBMC stocks by RT-PCR and PCR products were sequenced as described above.

### PBMC infection

PBMCs were collected from 18 macaques with TRIM5^TFP/TFP^ genotype and five with TRIM5^Q/Q^ genotype and activated with PHA as described above. 10^6^ activated PBMCs were infected with 10^3^ TCID_50_ 293T transfection virus stocks at 37°C for 60 minutes. The infected PBMCs were washed and cultured in complete RPMI 1640 media containing 10% IL-2. Virus production was monitored by reverse transcriptase (RT) activity of supernatant collected at 3-day intervals. RT values were quantified with Phosphor Imaging (FujiFilm, Japan). Replication curves were plotted based on RT values.

### Animal inoculations

Twelve STLV, SRV and SIV seronegative rhesus macaques with TRIM5^TFP/TFP^ genotypes were divided into two groups. The distribution of major histocompatibility complex (MHC) class I alleles Mamu-A*01, Mamu-B*08, and Mamu-B*17 were balanced between the two groups. Each macaque was inoculated intrarectally (I.R.) with a 1∶50 dilution of SIVsmE543-3 or SIVsmE543-3 S^37^S^98^ PBMC virus stocks (1000 TCID_50_, 5×10^5^ RNA copies). After inoculation, viral RNA levels in plasma were determined by quantitative RT-PCR. Four weeks later, any of the macaques that remained uninfected were inoculated intrarectally on a weekly schedule with same amount of virus until viral RNAs became detectable in plasma. After infection, blood and plasma were collected and plasma viral RNA levels were determined.

### Statistical analyses

All statistical analyses and graphic analyses were performed using GraphPad Prism5 (GraphPad Prism Software, La Jolla, CA). *In vitro* PBMC replication was assessed by Area under replication curves (AUC) calculation and pairwise compared by paired t test. Infectivity of SIV variants in the single cycle assay was calculated as a percentage of infectivity of the vector control in each cell line and compared statistically to the wild type using One Way ANOVA with Dunnett's Multiple Comparison Test. Kaplan Meier curves were plotted based on the inoculation number before infection and log-rank test was used to compare the acquisition of infection. Non-parametric Mann-Whitney-test was used for the comparison of viral loads between the two groups.

## Supporting Information

Figure S1 **Statistical comparison of SIVsmE543-3 and mutant replication on cell lines expressing different TRIM5 alleles (raw data shown in** **Fig. 4** **).** The replication of SIVsmE543-3 and its mutants on cell lines expressing TRIM5^TFP^ (A), TRIM5^Q^ (B) and TRIM5^TFP^ (C) alleles are showed as the percentage of the replication on cell line expressing vector control. Differences of replication between mutants and wild type SIVsmE543-3 were compared by one-way analysis of variance (ANOVA) with Dunnett's post-test. Pairs of groups that differed significantly are indicated (**,p<0.01, ***,p<0.001).
(TIF)
