# Supplementary figures and images for: TRIM5 alpha Drives SIVsmm Evolution in Rhesus Macaques
Source: PLoS Pathog. 2013 Aug 22;9(8):e1003577. doi: 10.1371/journal.ppat.1003577 (PMC3749954; doi:10.1371/journal.ppat.1003577)

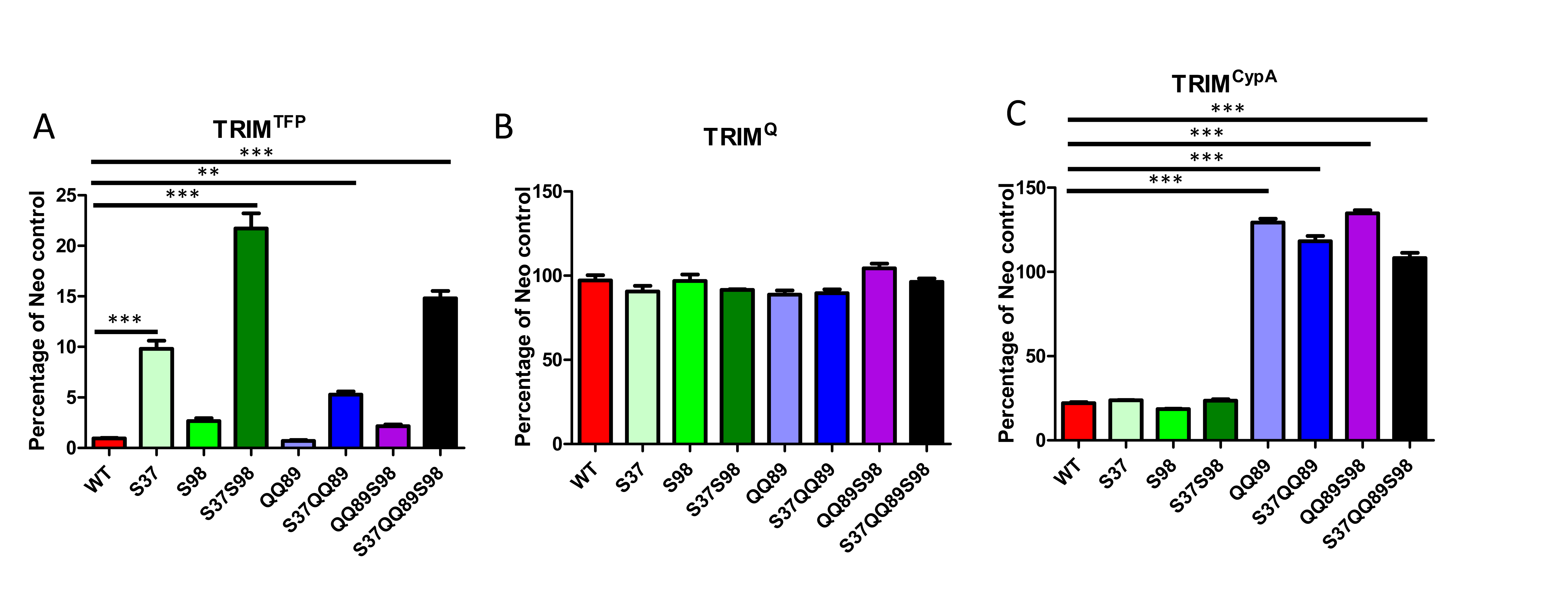

Supplement: Figure S1 — Statistical comparison of SIVsmE543-3 and mutant replication on cell lines expressing different TRIM5 alleles (raw data shown in Fig. 4 ). The replication of SIVsmE543-3 and its mutants on cell lines expressing TRIM5TFP (A), TRIM5Q (B) and TRIM5TFP (C) alleles are showed as the percentage of the replication on cell line expressing vector control. Differences of replication between mutants and wild type SIVsmE543-3 were compared by one-way analysis of variance (ANOVA) with Dunnett's post-test. Pairs of groups that differed significantly are indicated (**,p<0.01, ***,p<0.001). (TIF) [file ppat.1003577.s001.tif]
